# THE AGING NETWORK AND CLIMATE RESILIENCE

**DOI:** 10.1093/geroni/igad104.0843

**Published:** 2023-12-21

**Authors:** Holly Dabelko-Schoeny, Smitha Rao, Marisa Sheldon, White Katie

**Affiliations:** The Ohio State University, Columbus, Ohio, United States; The Ohio State University, Columbus, Ohio, United States; Ohio State University, Columbus, Ohio, United States; Central Ohio Area Agency On Aging, Columbus, Ohio, United States

## Abstract

Originally established to support planning and services related to community-based supports, Area Agencies on Aging (AAA) are well-positioned to lead cutting edge innovations driven by local community needs and changing socio-environmental contexts. This presentation illustrates a partnership between the Central Ohio Area Agency on Aging (COAAA) and the Age-Friendly Innovation Center at The Ohio State University focused on advancing research-informed practice in response to the realities of aging in a warmer world. This project included three main phases. First, safety and emergency preparedness data were collected from individuals aged 50 and older living in central Ohio through a random sample survey (N=1417), revealing 25% of non-white residents (compared to 21% white) did not have or know they have 3-day emergency supplies, food, and medicine, and 84% of residents in urban areas did not have access to an alternative source of power in event of outages. Second, a community conversation identifying priorities and needs related to the rising number of extreme weather events was held with COAAA leadership, community organizations, and residents. Third, targeting locations of high density of older adults at highest risk, the last phase of this work focused on the development of a tool to reduce harm for older adults living in affordable housing in partnership with COAAA embedded Service Coordinators. This project illustrates how AAAs can partner with applied researchers to innovate and strengthen services within communities and build evidence to address emerging community needs across the aging network.

